# Bilateral vocal cord palsy as complication of CNS tuberculosis

**DOI:** 10.1186/s12883-023-03308-1

**Published:** 2023-07-03

**Authors:** Liesa Regner-Nelke, Bendix Labeit, Christopher Nelke, Wolfram Schwindt, Rainer Dziewas, Sonja Suntrup-Krueger

**Affiliations:** 1 Department of Neurology, University Hospital Düsseldorf, Heinrich-Heine-University Düsseldorf, Moorenstraße 5, Düsseldorf, 40225 Germany; 2 Department of Neurology with Institute of Translational Neurology, University Hospital Münster, Albert-Schweitzer-Campus 1, Münster, 48149 Germany; 3 Department of Radiology, University Hospital Münster, Albert-Schweitzer-Campus 1, Münster, 48149 Germany; 4 Department of Neurology, Klinikum Osnabrück, Am Finkenhügel 1, Osnabrück, 49076 Germany

**Keywords:** Bilateral vocal cord palsy, Tuberculous meningitis, CNS tuberculosis, Cranial nerves, Case report

## Abstract

**Background:**

Tuberculous meningitis, a rare but severe form of extrapulmonary tuberculosis, frequently affects cranial nerves. While nerves III, VI and VII are commonly involved, involvement of caudal cranial nerves is rarely described. Here, we report a rare case of bilateral vocal cord palsy secondary to caudal cranial nerve involvement in tuberculous meningoencephalitis, that occurred in Germany, a country with low tuberculosis incidence.

**Case presentation:**

A 71-year-old woman was transferred for further treatment of hydrocephalus as a complication of presumed bacterial meningitis with unknown pathogen at that time. Because of decreased consciousness, intubation was performed and an empiric antibiotic therapy with ampicillin, ceftriaxone and acyclovir was initiated. Upon admission to our hospital, an external ventricular drainage was placed. Cerebrospinal fluid analysis revealed *Mycobacterium tuberculosis* as the causative pathogen, and antitubercular treatment was initiated. Extubation was possible one week after admission. Eleven days later, the patient developed inspiratory stridor that worsened within a few hours. Flexible endoscopic evaluation of swallowing (FEES) revealed new-onset bilateral vocal cord palsy as the cause of respiratory distress, which required re-intubation and tracheostomy. The bilateral vocal cord palsy persisted despite continued antitubercular therapy on the follow-up examination.

**Conclusion:**

Considering the aetiology of infectious meningitis, cranial nerve palsies may be suggestive for tuberculous meningitis as underlying disease given their rarity in other bacterial forms of meningitis. Nevertheless, intracranial involvement of inferior cranial nerves is rare even in this specific entity, as only extracranial lesions of inferior cranial nerves have been reported in tuberculosis. With this report of a rare case of bilateral vocal cord palsy due to intracranial involvement of the vagal nerves, we emphasize the importance of timely initiation of treatment for tuberculous meningitis. This may help to prevent serious complications and associated poor outcome since the response to anti-tuberculosis therapy may be limited.

## Background

Tuberculosis is an infectious disease caused by *Mycobacterium tuberculosis* (MTB) primarily affecting the lungs. Extrapulmonary manifestation, such as tuberculosis of the central nervous system (CNS), is possible. Tuberculous meningitis accounts for approximately 5–15% of extrapulmonary manifestations and thus can be considered a rare but severe form of manifestation [1].

Cranial nerve involvement is common in tuberculous meningitis and frequently presents as uni- or bilateral palsy, often affecting multiple nerves [2]. Most commonly, the cranial nerves III, VI and VII are affected [3]. In contrast, lesions of caudal cranial nerves are rarely described in CNS tuberculosis, and their prognostic value or response to treatment is understudied.

Tuberculosis is most common in sub-Sahara Africa, Eastern Europe and Asia [4].

In this case report, we describe an unusual case of a 71-year-old German woman who developed refractory acute bilateral vocal cord palsy during the course of tuberculous meningoencephalitis due to caudal cranial nerve involvement that persisted after initiation of antitubercular therapy.

## Case presentation

A 71-year-old woman was transferred from a regional hospital to our tertiary care clinic for further treatment of hydrocephalus as a complication of presumed bacterial meningitis with unknown pathogen. The patient had been admitted 7 days earlier for chronic exhaustion and weight loss. No immunocompromising pre-existing condition was known. During inpatient treatment, she had developed headache, fever and a progressive deterioration of consciousness. An examination of the cerebrospinal fluid (CSF) had revealed an elevated cell count (500 /µl, 4% polymorphonuclear cells; 97% mononuclear cells) with 495 leukocytes/µl (reference range: <5/µl) and an elevated protein level (7000 mg/l, reference range 150–450 mg/l)). Lactate was increased to 88.9 mg/dl (reference range: 10–22 mg/dl) and albumin to 4881 mg/dl (reference range: <350 mg/dl) while glucose level were reduced to 36 mg/dl (reference range: 40–70 mg/dl). Calculated anti-infective therapy with ampicillin, ceftriaxone and acyclovir had been started. Due to insufficient pulmonary protective reflexes and a further deterioration of consciousness, intubation was required 6 days after admission. A computed tomography (CT) scan of the brain had revealed occluding hydrocephalus with critical special condition of both hemispheres due to cerebral oedema. In addition, a CT scan of the thorax revealed a calcified pulmonary focus suggestive of a previous tuberculosis infection. Consequently, transfer to an external tertiary center for further treatment was organised.

**Fig. 1:**
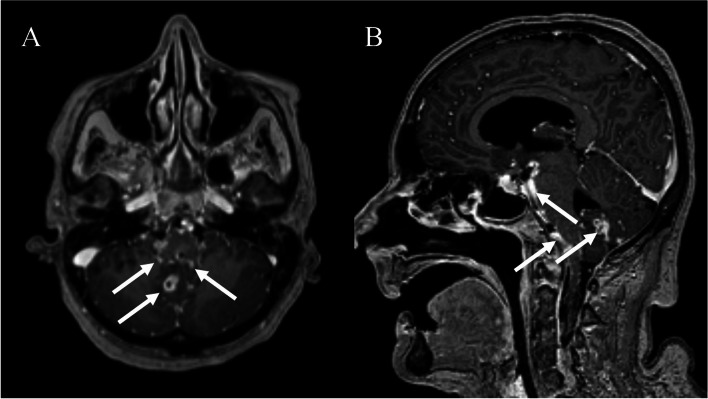
Contrast enhanced, T1-weighted MRI scan 35 days into treatment. **A** Axial section, showing several ring-shaped contrast enhancements (arrows) with a maximum diameter of 12 mm left cerebellar, right cerebellar and centrally in the cerebellar vermis. **B** Sagittal section showing leptomeningeal contrast enhancement (arrows), particularly in the cerebellum, medulla oblongata, and pons

On the day of admission at our neurological intensive care unit, an external ventricular drainage (EVD) was inserted to decrease the elevated intracranial pressure. Analysis of the ventricular CSF showed a decrease in cell count (40 cells/µl, reference range: <5/µl) and protein (3620 mg/l reference range 150–450 mg/l) compared to the previous analysis. Glucose remained reduced at 42.3 mg/dl (reference range: 49–75 mg/dl) and lactate elevated with 77,38 mg/dl (reference range: 15–23 mg/dl). Microbiological examination of the CSF by nucleic acid amplification test was performed detecting MTB as the causative pathogen. Antitubercular treatment with rifampicin, pyrazinamide, moxifloxacin and isoniazid was initiated the day after admission. The patient was treated with steroids at no time. A resistance testing to antitubercular therapy done in CSFs showed sensitivity for rifampicin, isoniazid, ethionamide. CSF cell count remained slightly elevated during the further course, and protein level was continuously elevated between 1000 and 3000 mg/l. One week after admission, the patient was successfully extubated due to improvement in consciousness. She was drowsy but able to fixate with her eyes when addressed and followed commands. Flexible endoscopic evaluation of swallowing (FEES) after extubation revealed moderate dysphagia with penetration of fluids but sufficient protective reflexes and normal laryngeal motor function. On day 19, the patient developed an inspiratory stridor, which worsened within a few hours. Vigilance remained stable. Further FEES now revealed bilateral vocal cord palsy as the cause of respiratory distress, requiring re-intubation and tracheostomy. A cranial magnetic resonance imaging (MRI) scan at day 35 revealed multiple linear parenchymal lesions, as well as micro-abscesses as signs of tuberculous meningoencephalitis (Fig. 1). Due to persistently high CSF outflow, which indicated malresorptive hydrocephalus, a ventriculoperitoneal shunt was surgically inserted during the further course. 64 days after admission, the patient was successfully weaned from ventilation but could not be decannulated because of persistent vocal cord palsy. Since adequate oral intake was not possible due to persistent drowsiness and compromised airways, a percutaneous endoscopic gastrostomy was performed on day 54. The final FEES before discharge on day 56 showed no pooling of secretions, a normal spontaneous swallowing frequency and intact laryngeal sensation. However, due to persistent bilateral vocal cord palsy, decannulation was not possible despite adequate swallowing function. Upon discharge, the patient was awake, fixated with her eyes, but did not follow commands. The patient was transferred to a neurorehabilitation facility for further therapy and rehabilitation. In the following months, anti-infective treatment was continued and the patient attended regular follow-up outpatient appointments in our department for neurosurgery to monitor the function of the ventriculoperitoneal shunt. At 4 months after discharge, FEES demonstrated persistent bilateral vocal cord palsy precluding decannulation.

## Discussion

When meningitis is suspected, cranial nerve palsies may be considered as indicative of tuberculous meningitis as the underlying disease, as they are rare in other bacterial meningitis [5]. In Germany, tuberculosis is rare with case numbers decreasing since 2017, leaving an incidence of 4.7 cases per 100,000 population in 2021 [6]. In low-incidence countries, such as Germany, most cases arise from reactivation of latent tuberculosis infections acquired abroad [7]. Thus, this is a rare cause of meningitis and is not usually included in routine diagnostics. Indeed, CNS tuberculosis, a common neurological disorder in developing countries, can lead to various complications, such as arteritis and phlebitis, leading to vasculitic infarcts due to arterial vasospasm, haemorrhage of the vessels, or thrombosis. An extension of the inflammatory process to the basal cisterns can result in hydrocephalus due to the hampering of CSF circulation and absorption. Furthermore, tuberculomas, presenting as deep-seated tubercular granulomatous foci, can occur. These can be clinically silent or result in tubercular abscesses [8]. Regarding cranial nerve palsies, the oculomotor nerve and abducens nerve are most commonly affected. Both nerves have a long path through the skull and are therefore prone to pathologic features of CSF. The pathophysiological hypotheses of cranial nerve involvement in tuberculous meningitis stems from a gelatinous exudate produced as an inflammatory response when MTB is released from subependymal or subpial granulomatous lesions into the subarachnoid space [9, 10]. This exudate, consisting of bacilli, erythrocytes and immune cells can encase nerves and vessels [11–13]. However, intracranial involvement of inferior cranial nerves is rare [14]. Only extracranial lower cranial nerve involvement, especially of the vagus nerve, e.g. in mediastinal or pharyngeal tuberculosis presenting as unilateral or bilateral vocal cord palsy, has repeatedly been reported [15–18].

In this case report, the bilateral vocal cord palsy was most likely the result of an intracranial vagal nerve lesion, as the CT scan of the neck and thorax did not show tuberculosis involvement of the vagal nerve in its extracranial course. To our knowledge, this has not been described before and is therefore an extremely rare cause of bilateral vocal cord paralysis in tuberculosis. Other courses of cranial nerve palsy, like vascular diseases (e.g. stroke), metabolic diseases (e.g. diabetes) and neoplasia had been excluded.

The vagus nerve, which innervates the laryngeal muscles responsible for opening the vocal cords, has only a short route through the skull before exiting through the jugular foramen and splitting into branches.

The bilateral vocal cord palsy described in this case required intubation and tracheostomy and resulted in prolonged intensive medical care. Interestingly, there was no improvement of the vocal cord palsy under anti-tuberculosis treatment. On the contrary, it occurred more than two weeks after the initiation of anti-infective therapy.

Since cranial nerve palsies are a common late sequel of tuberculous meningitis, poor response to anti-tuberculosis therapy has been described [19]. In contrast, case reports on mediastinal or pharyngeal tuberculosis resulting in vocal cord palsy describe adequate recovery after anti-tuberculous treatment [17, 18]. This case report demonstrates that cranial nerve impairment in tuberculous meningitis, particularly of the basal cranial nerve, is not only diagnostic but also prognostically important. Timely antibiotic treatment is crucial for patient outcome. Delayed treatment initiation (≥ 3 days) as well as an advanced clinical stage on admission are associated with a poor outcome [1, 20, 21]. In this case, the protracted diagnosis delayed the administration of appropriate treatment, resulting in an extremely complicated course of the disease.

## Conclusion

In summary, we report a rare and unusual case of bilateral vocal cord palsy in a German woman due to intracranial involvement of the vagal nerves. This case report highlights the importance of timely treatment initiation in tuberculous meningitis to prevent serious complications such as basal cranial nerve palsies, which may be associated with poor outcome since their response to antitubercular therapy is known to be poor.

## Data Availability

Not applicable.

## Abbreviations

CNS: Central nervous system
CSF: Cerebrospinal fluid
CT: Computed tomography
ENT: Ear, nose, throat
EVD: External ventricular drainage
FEES: Flexible endoscopic evaluation of swallowing
MRI: Magnetic resonance imaging
MTB: Mycobacterium tuberculosis
